# Supplementary material for: A common computational principle for vibrotactile pitch perception in mouse and human
Source: Nat Commun. 2021 Sep 9;12:5336. doi: 10.1038/s41467-021-25476-9 (PMC8429766; doi:10.1038/s41467-021-25476-9)
Supplement: Supplementary file 1 — Supplementary Information [file 41467_2021_25476_MOESM1_ESM.pdf]

## Supplementary Information

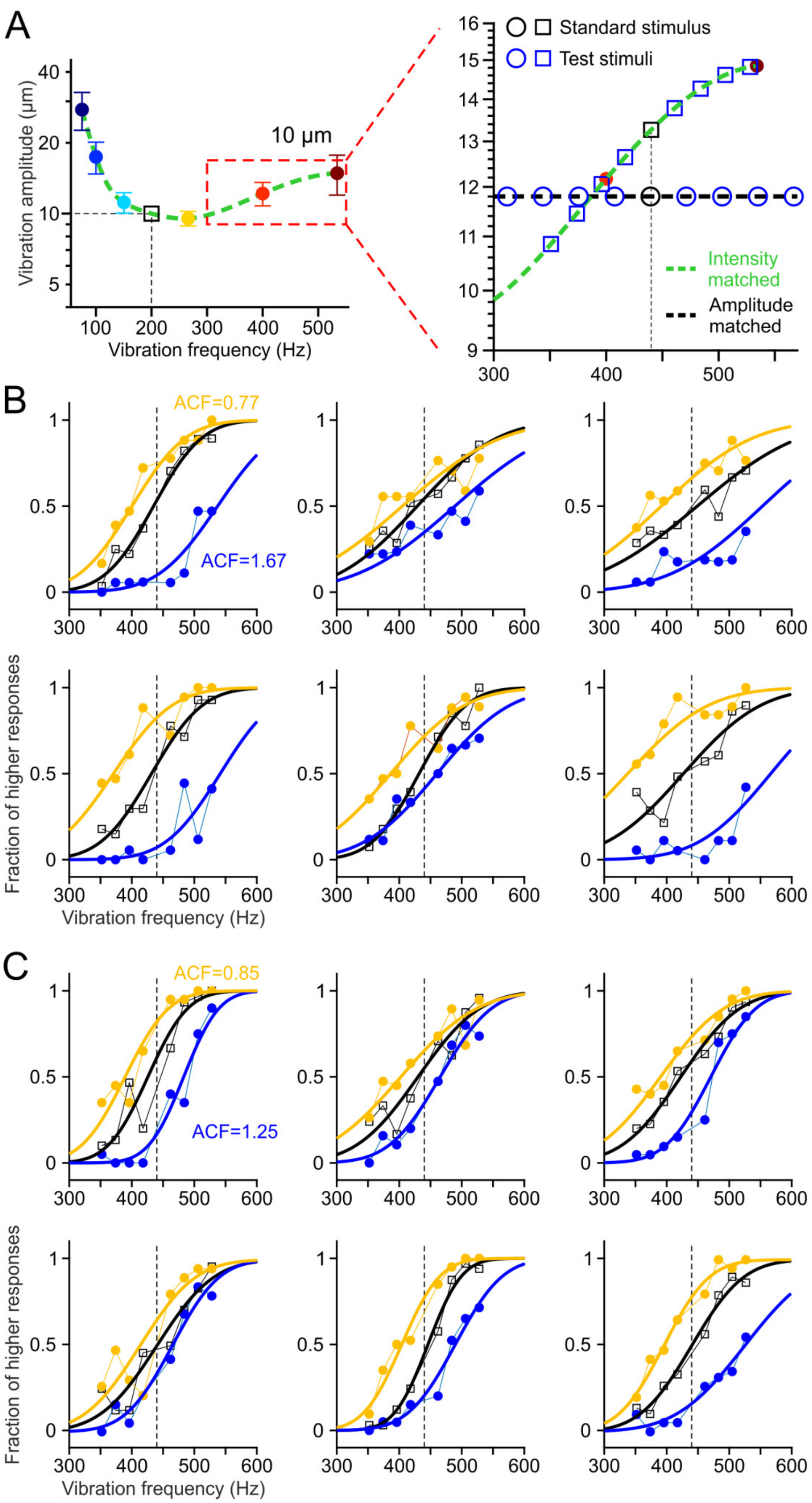

**Supplementary Figure 1.** Pitch discrimination along equal intensity curves. **A**: Eight test stimuli (blue squares) were compared in perceived pitch to a standard stimulus (black square). Their frequencies were uniformly distributed around the 440 Hz standard and their amplitudes were obtained from the 10 µm equal intensity curve of Fig. 4A (left panel, see Fig. 4A legend for details) meaning that all stimuli were matched in perceived intensity. Equal amplitude stimuli (circles) used in the experiment of Fig. 2 are shown for comparison. **B:** Psychometric curve fits (N=6 participants, individual panels) to the fraction of “higher” responses for vibrations with “matched” reference and test amplitudes (black), and for 2 tested amplitude change factors (yellow and blue). **C:** Same data as in B for intensity matched stimuli according to the 12 µm equal intensity curve of Fig. 4A. Source data are provided as a Source Data file.
