# Supplementary material for: A common computational principle for vibrotactile pitch perception in mouse and human
Source: Nat Commun. 2021 Sep 9;12:5336. doi: 10.1038/s41467-021-25476-9 (PMC8429766; doi:10.1038/s41467-021-25476-9)
Supplement: Supplementary file 3 — Reporting Summary [file 41467_2021_25476_MOESM3_ESM.pdf]

nature portfolio

Corresponding author(s): Mario Prsa, Daniel Huber

Last updated by author(s): Jul 20, 2021

Reporting Summary

Nature Portfolio wishes to improve the reproducibility of the work that we publish. This form provides structure for consistency and transparency in reporting. For further information on Nature Portfolio policies, see our Editorial Policies and the Editorial Policy Checklist.

Statistics

For all statistical analyses, confirm that the following items are present in the figure legend, table legend, main text, or Methods section.

n/a	Confirmed	
☐	☒	The exact sample size (n) for each experimental group/condition, given as a discrete number and unit of measurement
☐	☒	A statement on whether measurements were taken from distinct samples or whether the same sample was measured repeatedly
☒	☐	The statistical test(s) used AND whether they are one- or two-sided<br>*Only common tests should be described solely by name; describe more complex techniques in the Methods section.*
☐	☒	A description of all covariates tested
☒	☐	A description of any assumptions or corrections, such as tests of normality and adjustment for multiple comparisons
☐	☒	A full description of the statistical parameters including central tendency (e.g. means) or other basic estimates (e.g. regression coefficient) AND variation (e.g. standard deviation) or associated estimates of uncertainty (e.g. confidence intervals)
☒	☐	For null hypothesis testing, the test statistic (e.g. F, t, r) with confidence intervals, effect sizes, degrees of freedom and P value noted<br>*Give P values as exact values whenever suitable.*
☒	☐	For Bayesian analysis, information on the choice of priors and Markov chain Monte Carlo settings
☒	☐	For hierarchical and complex designs, identification of the appropriate level for tests and full reporting of outcomes
☒	☐	Estimates of effect sizes (e.g. Cohen's d, Pearson's r), indicating how they were calculated

Our web collection on statistics for biologists contains articles on many of the points above.

Software and code

Policy information about availability of computer code

Data collection	All data collection was performed with custom code in Matlab R2018a. The code is deposited in the YARETA database under accession code [https://doi.org/10.26037/yareta:p73yr7n7bnfnbp6wrt355g5q5e].
Data analysis	All data analysis was performed with custom code in Matlab R2018a. The code is deposited in the YARETA database under accession code [https://doi.org/10.26037/yareta:p73yr7n7bnfnbp6wrt355g5q5e].

For manuscripts utilizing custom algorithms or software that are central to the research but not yet described in published literature, software must be made available to editors and reviewers. We strongly encourage code deposition in a community repository (e.g. GitHub). See the Nature Portfolio guidelines for submitting code & software for further information.

Data

Policy information about availability of data

All manuscripts must include a data availability statement. This statement should provide the following information, where applicable:

- Accession codes, unique identifiers, or web links for publicly available datasets
- A description of any restrictions on data availability
- For clinical datasets or third party data, please ensure that the statement adheres to our policy

The data generated and/or analysed during the current study are deposited in the YARETA database under accession code [https://doi.org/10.26037/yareta:p73yr7n7bnfnbp6wrt355g5q5e].

Field-specific reporting

Please select the one below that is the best fit for your research. If you are not sure, read the appropriate sections before making your selection.

[x] Life sciences [ ] Behavioural & social sciences [ ] Ecological, evolutionary & environmental sciences

For a reference copy of the document with all sections, see nature.com/documents/nr-reporting-summary-flat.pdf

Life sciences study design

All studies must disclose on these points even when the disclosure is negative.

Sample size	Our study consists of a psychophysical quantification of a perceptual process. This quantification takes the form of a mathematical model fit to the data. Our study therefore does not involve any statistical tests comparing data between groups or conditions. A sample size determination procedure is therefore irrelevant for our study.
Data exclusions	For the frequency discrimination task in humans, 6 subjects were excluded from the analysis after realizing the actuator failed to generate vibrations at one of the amplitudes due to a coding error.
Replication	Our study does not involve any statistical tests comparing data between groups or conditions. Replication is therefore not applicable.
Randomization	No randomization was required as our study did not involve separating subjects into control and experimental groups.
Blinding	Our study did not involve separating subjects into control and experimental groups. Blinding during data collection with respect to group allocation is therefore not applicable for our study. Analyses of data comparing different experimental conditions in the same subjects were performed by blinded researchers.

Reporting for specific materials, systems and methods

We require information from authors about some types of materials, experimental systems and methods used in many studies. Here, indicate whether each material, system or method listed is relevant to your study. If you are not sure if a list item applies to your research, read the appropriate section before selecting a response.

Materials & experimental systems

n/a	Involved in the study	
[x]	[ ]	Antibodies
[x]	[ ]	Eukaryotic cell lines
[x]	[ ]	Palaeontology and archaeology
[ ]	[x]	Animals and other organisms
[ ]	[x]	Human research participants
[x]	[ ]	Clinical data
[x]	[ ]	Dual use research of concern

Methods

n/a	Involved in the study	
[x]	[ ]	ChIP-seq
[x]	[ ]	Flow cytometry
[x]	[ ]	MRI-based neuroimaging

Animals and other organisms

Policy information about studies involving animals; ARRIVE guidelines recommended for reporting animal research

Laboratory animals	All experiments were conducted with male and female C57BL/6 (Charles River Laboratory) mice, 10 to 20 weeks old at the start of behavioral training.
Wild animals	No wild animals were used.
Field-collected samples	The study did not involve field-collected samples.
Ethics oversight	All procedures involving mice complied with and were approved by the Institutional Animal Care and Use Committee of the University of Geneva and Geneva veterinary offices.

Note that full information on the approval of the study protocol must also be provided in the manuscript.

Human research participants

Policy information about studies involving human research participants

Population characteristics	The cohort included 24 participants aged between 21 and 48 years (mean ± s.d. = 29.25 ± 7.89, 12 females) with no history of somatosensory injury or disease, no psychiatric disorder and no substance abuse. Our study did not involve separating

	subjects into control and experimental groups. Description of covariant-relevant population characteristics (e.g. treatment categories) is therefore not applicable.
Recruitment	We recruited participants via flyers in universities and public places. Key inclusion criteria were the age (18 to 50 years old) and normal or corrected-to-normal vision. We excluded all participants with a history of psychiatric disorders, with a Median or Ulnar nerve damage causing numbness in the finger tips, and the use of any medication causing insensitivity. We do not identify any potential selection bias.
Ethics oversight	All experimental procedures involving human participants were approved by the ethics commission of the Geneva canton (CCER, Cantonal Swiss Ethics Committee on research involving humans), University of Geneva.

Note that full information on the approval of the study protocol must also be provided in the manuscript.
